# PETAL LOSS and ROXY1 Interact to Limit Growth Within and between Sepals But to Promote Petal Initiation in *Arabidopsis thaliana*

**DOI:** 10.3389/fpls.2017.00152

**Published:** 2017-02-08

**Authors:** Tezz Quon, Edwin R. Lampugnani, David R. Smyth

**Affiliations:** School of Biological Sciences, Monash University, ClaytonVIC, Australia

**Keywords:** AUX1, flower development, glutaredoxin, petal, PETAL LOSS, ROXY1, sepal, trihelix

## Abstract

The activity of genes controlling organ development may be associated with the redox state of subregions within the meristem. Glutaredoxins react to the level of oxidative potential and can reduce cysteine dithiols, in some cases to activate specific transcription factors. In Arabidopsis, loss of function of the glutaredoxin ROXY1 or the trihelix transcription factor PETAL LOSS (PTL) each results in reduced numbers of petals. Here, genetic studies have revealed that loss of petals in *ptl* mutant plants depends on ROXY1 function. The two genes also act together to restrain stamen-identifying C function from entering the outer whorls. On the other hand, they suppress growth between sepals and in sepal margins, with ROXY1 action partially redundant to that of PTL. Genetic interactions with *aux1* mutations indicate that auxin activity is reduced in the petal whorl of *roxy1* mutants as in *ptl* mutants. However, it is apparently increased in the sepal whorl of triple mutants associated with the ectopic outgrowth of sepal margins, and of finger-like extensions of inter-sepal zones that in 20% of cases are topped with bunches of ectopic sepals. These interactions may be indirect, although PTL and ROXY1 proteins can interact directly when co-expressed in a transient assay. Changes of conserved cysteines within PTL to similar amino acids that cannot be oxidized did not block its function. It may be in some cases that under reducing conditions ROXY1 binds PTL and activates it by reducing specific conserved cysteines, thus resulting in growth suppression.

## Introduction

Meristems are the ultimate source of all plant tissues. Primary meristems are characterized by a core of slowly dividing stem cells surrounded by regions of growth, organogenesis, and eventually cellular differentiation (Sablowski, 2010). In shoot and flower meristems, cell division increases in the peripheral zone surrounding the stem cells, and organ initiation is signaled within this zone at defined times and locations. Specific transcription factors and signal molecules have been uncovered in Arabidopsis that maintain the stem cell population, especially members of the homeodomain WUSCHEL (WUS) family in association with CLAVATA (CLV) signaling molecules (Schoof et al., 2000). Another homeodomain transcription factor, SHOOT MERISTEMLESS (STM), maintains the undifferentiated peripheral zone, with its function fading as organ primordia develop (Long et al., 1996), regulated by auxin dynamics (Reinhardt et al., 2003). Boundaries between developing organ primordia are reinforced by dampening of cell divisions associated with expression of *CUP-SHAPED COTYLEDON* (*CUC*) genes of the NAC transcription factor family (Aida et al., 1997). More recent understanding of mechanisms of meristem development has built on these foundation studies (for reviews, see Žádníková and Simon, 2014; Hepworth and Pautot, 2015; Galli and Gallivotti, 2016).

Cell division in subregions of meristems may be correlated with their oxidative state (Rouhier et al., 2015; Schippers et al., 2016). For example, the quiescent center of root meristems, with low cell division rates, is associated with low oxygen (reducing) conditions (Jiang et al., 2003). In this case, glutathione, a tripeptide that functions to modulate the reduced state of thiols in proteins, is required as its disruption by mutation results in loss of the root quiescent center, and stalling of cell division at the G1 to S phase transition (Vernoux et al., 2000). Inflorescence and flower meristem development is also moderated by redox status associated with glutathione function (Bashandy et al., 2010).

A key process that may respond to redox conditions is the regulation of transcription factor activity (Dietz, 2014; Schmidt and Schippers, 2015). For example, covalent disulfide bonds between two cysteine residues may need to be reduced for DNA binding to occur. In plants, this has been shown for R2R3 Myb (Heine et al., 2004), class 3 HD-ZIP (Comelli and Gonzalez, 2007), AP2 (Shaikhali et al., 2008), TCP (Viola et al., 2013), and bZIP (Shaikhali et al., 2012; Gutsche and Zachgo, 2016) family members. Even so, in most cases whether it also occurs *in vivo* to regulate specific responses to redox conditions has not been established.

One class of oxidoreductases that catalyze the modification of protein thiol groups dependant on oxygen availability are the glutaredoxins (GRXs) (Ströher and Millar, 2012; Gutsche et al., 2015). These have a conserved active site where a key cysteine is directly involved in the reduction of disulfide bonds to unconjugated thiol (SH) groups, usually involving glutathione. In plants, there are three sub-classes of GRXs, defined by their active site. The largest of these have adjacent cysteines and are known as CC GRXs (Ziemann et al., 2009; Gutsche et al., 2015). Their biological functions are being revealed by biochemical and genetic experiments, including responses to oxidative stress and regulation of specific developmental decisions. The first GRX shown to be involved in development was identified through analysis of *roxy1* mutants of Arabidopsis (Xing et al., 2005). These mutants have reduced numbers of petals, with many of the remaining ones being folded or smaller than normal. There are 21 CC GRX family members in Arabidopsis and they have been named ROXY1-21 depending on their degree of relatedness to ROXY1 (Li et al., 2009).

Some ROXY family members interact with bZIP transcription factors of the TGA class (Després et al., 2003). For example, ROXY1 and 2 bind to PERIANTHIA (PAN, also named TGA8), TGA9 and TGA10 *in vitro* and in yeast (Li et al., 2009). PAN is involved in defining the floral blueprint of the Arabidopsis flower, with five sepals and five petals often present in *pan* mutants instead of the normal four (Running and Meyerowitz, 1996; Chuang et al., 1999). Genetic interactions suggest that ROXY1 negatively regulates PAN’s inhibition of petal initiation. Whether this occurs through ROXY1 controlling the redox state of cysteines in PAN has not been established, although recent evidence shows that PAN can bind its specific DNA target *in vitro* only under reducing conditions (Gutsche and Zachgo, 2016).

Petal initiation is also disrupted in mutants of the *PETAL LOSS* (*PTL*) gene (Griffith et al., 1999). PTL encodes a transcription factor of the trihelix family (Brewer et al., 2004), and its main role in the flower is to repress growth in the sepal whorl rather than in the petal region where it is not expressed. In *ptl* mutants there are some additional cell divisions between sepals, and the sepals themselves are wider and deeper in profile, defects that correlate with the early expression pattern of *PTL* at these two locations (Brewer et al., 2004; Lampugnani et al., 2012). The disruption of petal initiation in *ptl* mutants, however, may be indirect and a consequence of growth-induced distortions to auxin accumulation internal to the sepal whorl (Lampugnani et al., 2013). We showed this by generating auxin in the inter-sepal zone where PTL is normally expressed, and restoring nearby petal initiation in *ptl* loss-of-function mutants. Also, residual petals that arise in *ptl* single mutant flowers were lost when auxin accumulation into the epidermis, controlled by the auxin influx permease AUXIN1 (AUX1) (Bennett et al., 1996), was jointly compromised. Disruption of polar auxin transport, controlled by PINOID (PID) and PIN-FORMED1 (PIN1), had similar consequences (Lampugnani et al., 2013).

In this study, we were interested to determine if PTL and ROXY1 functions overlap in floral organogenesis. Mutant and mis-expression approaches revealed that ROXY1 supports the disruption of petal initiation seen in *ptl* mutants. Further, the two proteins act in combination to prevent stamen-identifying C function from moving into the second whorl. By introducing the *aux1* mutant we revealed that ROXY1 and PTL together maintain appropriate auxin activity in the petal initiation zone. On the other hand, mutant defects in the sepal whorl indicated that these are caused by the release of normal growth inhibition, at least partly associated with heightened, not reduced, auxin function. This occurred to such an extent that when PTL, ROXY1, and AUX1 functions were all lost, inter-sepal zones often generated stalked outgrowths capped by clusters of sepals independent of the normal sepals. We further showed that PTL and ROXY1 proteins can bind following transient co-expression, and that three conserved cysteines in PTL can be changed to serine or alanine (and thus incapable of forming cysteine bridges) without losing petal initiation function. This is consistent with PTL acting when the cysteines are in a reduced state, although whether this is controlled by ROXY1 requires further study.

## Materials and Methods

### Origin of Arabidopsis Mutant Lines and Scoring of Flowers

The *ptl-1* mutant, in Columbia background (Griffith et al., 1999), is a null mutation encoding a premature stop at codon 120 (Brewer et al., 2004). The *ptl-3* mutant in Landsberg *erecta*, used in one experiment, carries a 100 bp deletion from codon 216 (Brewer et al., 2004). The *roxy1-3* null mutant, in Columbia, has a T-DNA insertion in the coding region (Xing et al., 2005), and was obtained from the Arabidopsis Biological Resources Center (ABRC) (CS328164; GABI-Kat GK-268A11.01). The *aux1-7* and *aux1-21* mutants, also in Columbia, were sourced from ABRC (CS3074, CS9584). *aux1-21* is a null mutation carrying an X-ray-induced deletion of one nucleotide leading to a premature stop at codon 279, and *aux1-7* is a partial loss of function point mutation (G459D) (Swarup et al., 2004).

Multiple mutants were generated by inter-crossing single or double mutant plants, and required homozygous individuals identified in the resulting F2 families by PCR analysis (**Supplementary Table S1**). F3 progeny plants of relevant genotypes were grown together, either at 20°C with 16 h of Cool White fluorescent light, or in a glasshouse at ∼20–25°C, with natural daylight supplemented by continuous fluorescent light. The first 10 flowers of the primary inflorescences of seven or eight F3 plants were scored for each genotype, with the number and form of organs in all four whorls recorded. Comparisons of petal numbers were based solely on plants grown together as this phenotype in *ptl* mutant plants is sensitive to environmental conditions, especially temperature (Griffith et al., 1999). In some cases, siliques were imaged by scanning electron microscopy (SEM) directly without fixation using a Quanta FEG 200 ESEM, or using either a Hitachi s570 microscope following glutaraldehyde fixation (Griffith et al., 1999).

### Modification of Intra-cellular Localization (MILo) Procedure

To test if the PTL and ROXY1 proteins can associate, the MILo method was used (Kaplan-Levy et al., 2014). To generate 35S:YFP-ROXY1 and 35S:YFP-ROXY1ΔC129 (a C-terminal deletion of eight codons), ROXY1 coding sequence was amplified from genomic DNA (*ROXY1* is intron-free) (see **Supplementary Table S1** for primers) and cloned behind YFP coding sequences that had been inserted in pART7 (Kaplan-Levy et al., 2014). The construction of 35S:CFPN7-PTL, in which mCerulean (CFP) was translationally fused with the strong nuclear-localization sequence N7, has been described (Kaplan-Levy et al., 2014). In the converse arrangement, cytoplasmic 35S:YFP-PTLmNLS (with mutant nuclear localization sequences) was from Kaplan-Levy et al. (2014), and nuclear 35S:CFPN7-ROXY1 was constructed by inserting the amplified ROXY1 genomic sequence into pART7 already containing CFPN7 downstream of the 35S promoter. The expression cassettes were excised with *Not*I, inserted into pMLBART and the plasmids electroporated into *Agrobacterium tumefaciens* strain AGL1.

Cultures of the two components were mixed and inoculated into maturing leaves of *Nicotiana benthamiana* in duplicate (Kaplan-Levy et al., 2014). After 3–4 days the inoculated sectors were cut out, mounted in water, and the lower surface visualized using a Zeiss Axiophot 2 mot plus microscope, with images collected using AxioVision software. YFP fluorescence was observed using filter set 46 (excitation BP500-20; beam splitter FT515; emission BP535/30), and CFP with filter set 4 (excitation BP470-20; beam splitter FT495; emission BP505-530). YFP fluorescence showed no overlap with CFP fluorescence and was used to assess protein-protein interactions. All MILo experiments were repeated on three or more occasions, and always included controls of each construct inoculated alone, and combinations in which the 35S:CFPN7 construct lacked the translationally fused “bait” protein.

### Generation of Constructs and Transgenic Plants, and Complementation Tests

To test the functions of ROXY1 or PTL in petal initiation, each was expressed in mutant plants under the control of the PTL promoter region, pPTL(1.3i) (Brewer et al., 2004; Lampugnani et al., 2012). This included 1.3 kbp upstream of the transcription start site, the 5′ UTR, the first exon and intron, and eight codons of the second exon. This can fully complement *ptl-1* mutant plants when driving expression of full-length PTL (Brewer et al., 2004).

The promoter was attached to a cassette containing a GUS reporter flanked by the self-cleavage peptide sequence T2A to release the GUS protein [pPTL(1.3i):2A-GUS-2A] (Kaplan-Levy et al., 2014). The gene to be tested, either ROXY1 or PTL (**Supplementary Table S1**) was then translationally fused to this and inserted in pMIGRO (Lampugnani et al., 2013), a derivative of pBJ36 (Gleave, 1992). The cassettes were excised by *Not*I and inserted in pMLBART as above. Site-directed mutagenesis of cysteines in the PTL protein was performed using a two-step PCR procedure with two divergent primers encoding the required substitution (**Supplementary Table S1**) (Kaplan-Levy et al., 2014).

Mutant plants were transformed and transgenic T1 plants selected by Basta resistance. T-DNA inserts were confirmed by PCR using the GUS+1550 forward primer and an internal gene reverse primer, or the 3′ OCS reverse primer for the empty vector (negative control) (**Supplementary Table S1**). Twelve to fifteen T1 plants chosen at random for each construct and scored for complementation of second whorl organ number in the first 10 flowers of the main inflorescence. If there was no complementation, five or more plants were stained for GUS product (Brewer et al., 2004) to confirm expression of the pPTL(1.3i):2A-GUS-2A cassette. All comparisons between constructs within an experiment were grown and scored together (see above).

## Results

### ROXY1 Promotes the Reduction in Petal Initiation that Occurs in *ptl* Mutant Flowers

**FIGURE 1:**
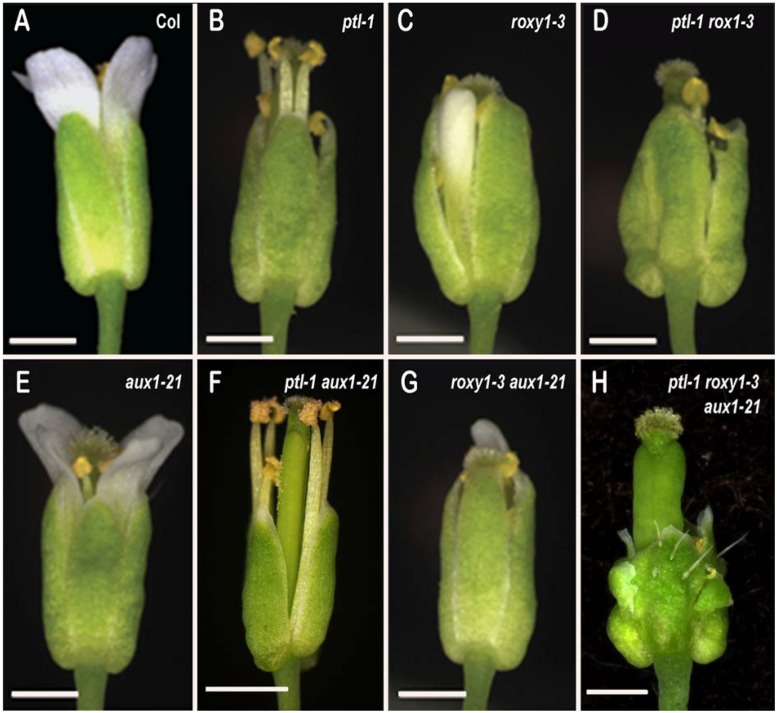
**Flowers of Arabidopsis carrying all combinations of *ptl-1. roxy1-3* and *aux1-21* mutations.** **(A)** Wild type Columbia. **(B)** *ptl-1*. **(C)** *roxy1-3*. **(D)** *ptl-1 roxy1-3*. **(E)** *aux1-21*. **(F)** *ptl-1 aux1-21*. **(G)** *roxy1-3 aux1-21*. **(H)** *ptl-1 roxy1-3 aux1-21*. Bar represents 0.5 mm

**FIGURE 2:**
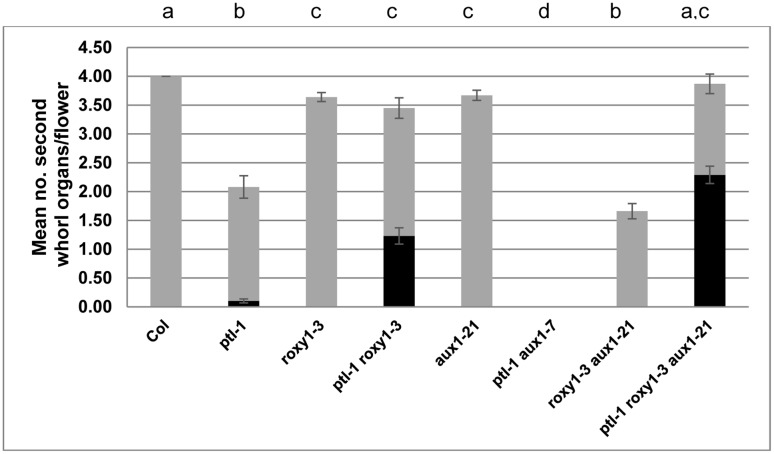
**Mean number of second whorl organs per flower in combinations of *ptl-1. roxy1-3*, and *aux1* mutations.** All plants were grown together, and the first 10 flowers on the main inflorescence scored (*N* = 10). The mean number of all second whorl organs is shown, with stamenoid petals and stamens highlighted in black. The *aux1-21* allele was used except for the *ptl-1 aux1-7* combination where only *aux1-7* was available. [In another experiment *ptl-1 aux1-21* also generated 0.00 second whorl organs (Lampugnani et al., 2013)]. Bars represent standard error of the mean (SEM). Pairwise comparisons by *t-*tests were carried out. Observations that were not significantly different (*p* > 0.05) are indicated by the same letter at the top of each column. All other comparisons were significantly different (*p* < 0.01).

Loss of either PTL or ROXY1 function results in fewer second whorl organs being formed (Griffith et al., 1999; Xing et al., 2005), although the disruption is weaker in *roxy1* mutants. To test if the processes involved are shared by the two genes, *ptl-1 roxy1-3* double mutants were observed (**Figures 1A–D**). This revealed that *roxy1-3* was epistatic to *ptl-1* in the number of second whorl organs formed. In one experiment, this was now 3.45 ± 0.178 compared with 2.08 ± 0.195 in *ptl-1* single mutants, but close to 3.64 ± 0.077 found in *roxy1-3* single mutants (**Figure 2**; **Supplementary Table S2**). Thus the relatively severe loss seen in *ptl-1* mutants is dependent on ROXY1 function.

Although the number of second whorl organs was similar in *roxy1-3* and *ptl-1 roxy1-3* mutant flowers, their identity was affected in the double mutant plants (**Supplementary Table S3**). More than one third were now stamens or stamenoid petals, not seen in *roxy1-3* single mutants and only occasionally in *ptl-1* (**Figure 2**). Significant numbers of filamentous organs arose as well. The stamens were not present at the expense of third whorl stamens, as these occurred in *ptl-1 roxy1-3* double mutants at the same levels as in *roxy1-3* single mutants (**Supplementary Table S4**). It seems that the boundary of C function, which together with B function confers stamen identity in the third whorl, is often extended outward into the second whorl if the functions of PTL and ROXY1 are both disrupted. The numbers of sepals and carpels were unaffected (**Supplementary Table S2**).

**FIGURE 3:**
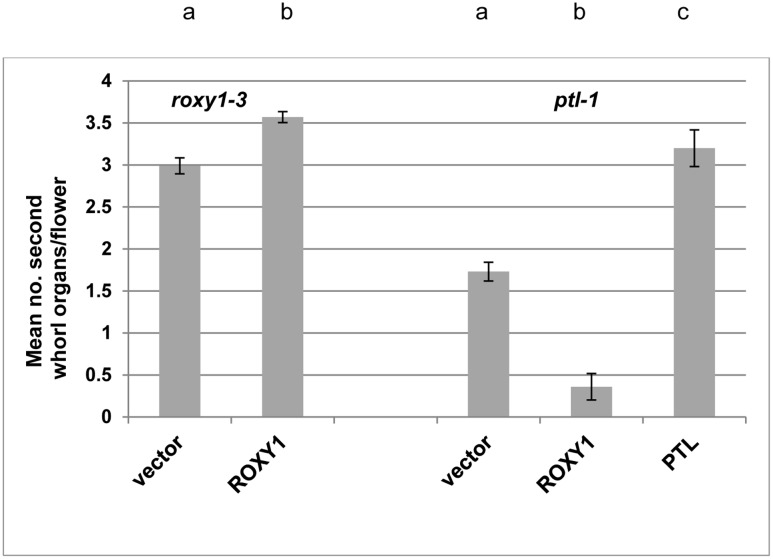
**Effect of ectopically expressing *ROXY1* in the *PTL* expression zone on second whorl organ development.** Mean number of second whorl organs per flower in *roxy1-3* (left) or *ptl-1* (right) T1 plants transformed with ROXY1 or PTL under the control of the PTL promoter region and downstream of a GUS-encoding sequence flanked by self-cleavage peptide sequences 2A that upon translation result in excision of both GUS and the downstream protein [pPTL(1.3i):2A-GUS-2A]. Negative controls involved T1 plants transformed with the empty vector, and positive controls involved *roxy-1* plants transformed with ROXY1, and *ptl-1* plants transformed with PTL. Individual data for the 8–15 T1 plants scored for each treatment are provided in **Supplementary Figure S2**. Bars represent SEM. Pairwise comparisons by *t*-tests were carried out. All comparisons were significantly different (*p* < 0.01) as indicated by the different letters at the top of each column for the *roxy1-3* transformants (left), and independently for the *ptl-1* transformants (right).

We next tested if the provision of ectopic ROXY1 activity could further decrease second whorl organ number in *ptl* mutant flowers. To do this ROXY1 was generated in the PTL expression domain, namely in the inter-sepal zone at stages 3 and 4, and in sepal margins at stage 4 (Smyth et al., 1990) (**Supplementary Figure S1**). Transgenic plants carrying pPTL(1.3i):2A-GUS-2A-ROXY1 were made (**Figure 3**; **Supplementary Figure S2**). Firstly, we showed that there was no detectable effect on petal number in transformed wild type controls. Then to test if the *ROXY1* transgene was active, we transformed it into *roxy1-3*. Second whorl organ numbers were significantly boosted, confirming its activity (**Figure 3**). In transgenic *ptl-1* mutant plants, however, the ROXY1 construct had a striking consequence, with very few second whorl organs now present. The mean number per flower was only 0.36 ± 0.157 compared with 1.73 ± 0.112 in empty vector controls. This supports the conclusion that, in the absence of PTL function, ROXY1 acts to decrease organ initiation in the second whorl.

### Epidermal Auxin Influx Promotes Initiation of Second Whorl Organs When ROXY1 Function Is Lost

We have earlier shown that the promotion of organ initiation in the second whorl by auxin is supported by PTL function. The AUX1 protein encodes an auxin influx channel (Bennett et al., 1996) confined to the epidermal (L1) layer (Reinhardt et al., 2003), and if its function is lost in *ptl* mutant background almost no petals now arise (Lampugnani et al., 2013) (**Figures 1E,F** and **2**). To test if loss of ROXY1 function has a similar effect, *roxy1-3 aux1-21* double mutants were generated (**Figure 1G**). Again, the number of second whorl organs was significantly reduced, although the loss was not as severe (**Figure 2**). The mean number per flower (1.66 ± 0.133) was around half of that recorded in *roxy1-3*. (In *aux1-21* single mutants only an occasional petal was lost.) There were no identity changes, although small and/or folded petals were frequent as observed in *roxy1-3* single mutants (**Supplementary Table S3**).

### Epidermal Auxin Influx Is Not Required for Initiation of Second Whorl Organs When Both PTL and ROXY1 Functions Are Compromised

As loss of either PTL or ROXY1 function alone increases the sensitivity of second whorl organ initiation to auxin disruption, we examined the consequence of losing both when auxin influx was also compromised. Triple *ptl-1 roxy1-3 aux1-21* mutants were generated (**Figure 1H**) and the surprising result was that second whorl organ numbers remained high, and were not significantly different from *ptl-1 roxy1-3* double mutant flowers (**Figure 2**). Their identity, too, was still affected (**Figure 2**; **Supplementary Table S3**). Indeed, stamens and stamenoid petals were even more frequent in *ptl-1 roxy1-3 aux1-21* triple mutant flowers (2.29 ± 0.151 per flower) than in *ptl-1 roxy1-3* doubles (1.23 ± 0.142) (*t* = 5.11, df = 154, *p* < 0.0001). Thus simultaneous loss of both PTL and ROXY1 functions somehow relieves the second whorl from sensitivity to reduced auxin influx into the epidermis.

### PTL and ROXY1 Function together to Limit Growth in the First Whorl

**FIGURE 4:**
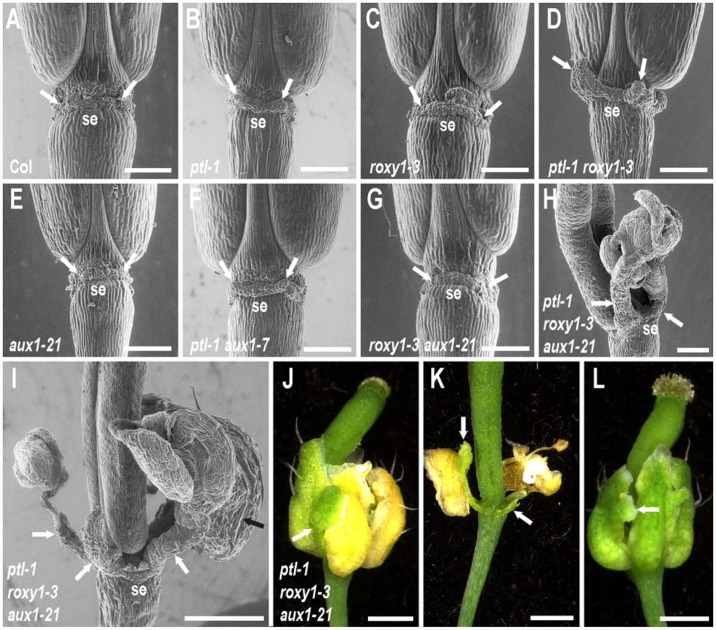
**Role of PTL and ROXY1 in limiting growth within the first whorl.** **(A–I)** SEMs of the base of mature stage 17 siliques of wild type and combinations of *ptl-1. roxy1-3*, and *aux1* mutations in lateral view, with the site of abscission of one sepal labeled below (se) and the two adjacent inter-sepal zones indicated by white arrows. Some outgrowth of the inter-sepal zone occurred in *ptl-1* single mutant siliques (arrows in **B**) compared with the same region in wild type controls (arrows in **A**). This was enhanced in *ptl-1 roxy1-3* double mutants **(D)**, but did not occur in *roxy1-3* single mutants **(C)**. Addition of *aux1* mutation had no effect alone **(E)** or in combination with *roxy1-3* **(G)**, and did not result in further outgrowth with *ptl-1* **(F)**. However, greatly enhanced outgrowth often occurred in *ptl-1 roxy1-3 aux1-21* triple mutants where clusters of ectopic organs on stalks may arise (white arrows in **H**, **I**). These were sepal-like, with characteristic long cells on their abaxial surface (black arrow in **I**). **(J–L)** Light micrographs of flowers of a *ptl-1 roxy1-3 aux1-21* triple mutant plants. Outgrowths of inter-sepal zone tissues (arrows in **J**, **K**) senesced later than sepals and remained green after the normal sepals had turned yellow **(J)** and later abscised **(K)**. Ectopic growths on the margins of the normally placed sepals was often observed, including additional sepal-like tissue (**L**, arrow). All plants were grown together and are from the same experiment shown in **Figure 2**. Bars represent 250 μm **(A–H)** or 500 μm **(I–L)**.

Turning to the first whorl, PTL functions to dampen growth of sepal margins, and of radial outgrowth between sepals. In *ptl* single mutants, mature sepals are larger and deeper in cross-section (Griffith et al., 1999), and the inter-sepal zone of stage 4 flowers is radially enlarged by 35–40% (about one cell’s width) (Lampugnani et al., 2013). Here, we have extended this latter observation to mature green siliques (stage 17) where the region between the scars of the now-abscised sepals in *ptl-1* mutants is still enlarged (**Figures 4A,B**). This was also observed in *ptl-3* mutants in Landsberg *erecta* background (**Supplementary Figure S3**).

ROXY1 function is also involved with PTL in first-whorl growth suppression. In siliques, outgrowth of the inter-sepal zone was further extended, with small, finger-like projections generated at each position in *ptl-1 roxy1-3* double mutants (**Figure 4D**). Sepals, too, were further enlarged compared with *ptl-1* single mutants (**Figure 1D**). Their surface was more buckled, and their edges more uneven. These excess growth patterns were not apparent in *roxy1-3* single mutants (**Figures 1C** and **4C**), so loss of PTL function is necessary for this suppressive role of ROXY1 to be revealed.

### PTL and ROXY1 Together Limit Auxin Function in the First Whorl

We next showed that movement of auxin into the epidermis plays a role in keeping extra first whorl growth in check (**Figures 1E–H**). In *ptl-1 roxy1-3 aux1-21* triple mutants, striking outgrowths now frequently occurred from the inter-sepal zones (**Figures 4H–K**). These were capped by clusters of 1–7 ectopic sepalloid organs (mode was three organs), some on an obvious stalk, in almost half (38 out of 78) of the flowers scored (e.g., **Figures 4H,I**). The ectopic organs were predominantly sepal-like, with long cells characteristic of sepals present on their abaxial surface (**Figure 4I**), although they sometimes showed marginal outgrowths of stigmata and ovule-like organs (in 6 out of 78 flowers). Overall, 19.9% of inter-sepal zones showed such additional organogenesis in triple mutants (**Supplementary Table S6**). The outgrowths were not joined to the adjacent flanking sepals, and when the latter senesced and abscised the inter-sepal extensions remained green and attached to the receptacle (**Figures 4J,K**). Ectopic outgrowths from the inter-sepal zone were also seen in *ptl-1 roxy1-3* double mutant flowers, although less frequently (in 14 out of 78 flowers, 5.8% of inter-sepal zones) (**Supplementary Table S6**), and only up to three sepal-like organs occurred in the clusters.

In *ptl-1 roxy1-3 aux1-21* triple mutants, overgrowth of the four normally located sepals was also more extensive than in *ptl-1 roxy1-3* doubles, and they were frequently buckled and folded (**Figures 1H** and **4L**). Also they often showed ectopic outgrowths along their margins, especially of sepal-like tissue (an average of 0.269 ± 0.081 times per flower) (**Figure 4L**) or carpelloid tissues (0.615 ± 0.145 per flower), the later including stigmata and ovule-like organs (**Supplementary Table S5**). Carpelloid tissues were also seen on the edges of sepals in single or double mutant plants that carry *ptl-1*, although much less often (on 0.026 to 0.054 occasions per flower) (**Supplementary Table S5**).

Simultaneous loss of both PTL and ROXY1 function was required to reveal this role for auxin influx. When *aux1* was combined with either *ptl-1* or *roxy1-3* individually, inter-sepal zone extension and sepal size were not distinguishably different from the single mutants (**Figures 1F,G** and **4E–G**).

Thus outgrowth and differentiation of the spaces between sepals, and the expansion of the sepals themselves, are each boosted when PTL and ROXY1 functions are jointly compromised, and further loss of AUX1 function results in much stronger ectopic growth.

### PTL and ROXY1 Proteins Interact When Transiently Expressed in Leaves

To test if ROXY1 can interact directly with PTL, we used the MILo (Modification of Intra-cellular Localization) method (Kaplan-Levy et al., 2014). In this, one protein, localized to the cytoplasm, was tagged with YFP, whereas the other was nuclear localized and tagged with CFP. They were transiently expressed together in leaves of *Nicotiana benthamiana*, and if preferential nuclear accumulation of YFP now occurred, we conclude that the two proteins bind with high affinity.

**FIGURE 5:**
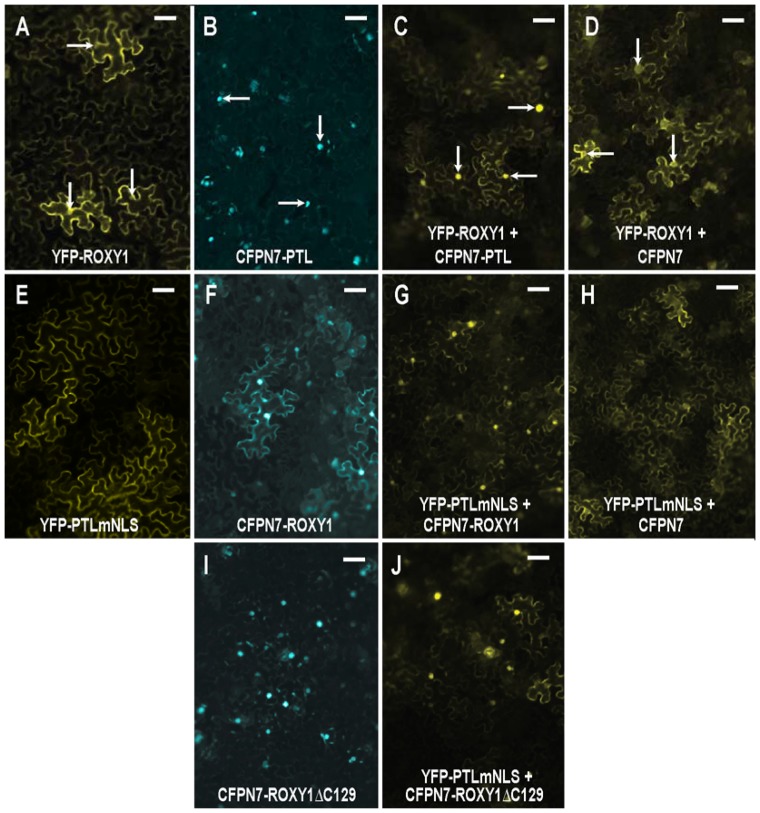
**Interaction between PTL and ROXY1 proteins following transient expression in leaves of *Nicotiana benthamiana.*** **(A–D)** ROXY1 tagged with YFP accumulated in the cytoplasm, with some faint nuclear fluorescence **(A)** unless co-expressed with PTL tagged with the nuclear-localized CFPN7 **(B)**, in which case significant nuclear localization of YFP-ROXY1 now occurred **(C)**. This did not occur when ROXY1 was co-expressed with CFPN7 sequences lacking PTL **(D)**. Arrows indicate nuclei in **(A–D)**. **(E–H)** PTLmNLS tagged with YFP was cytoplasmic owing to mutation of the three NLSs **(E)**, unless co-expressed with CFPN7-ROXY1 **(F)**, in which case the YFP-PTLmNLS product was now significantly nuclear **(G)**. Controls involving CFPN7 lacking ROXY1 were not effective **(H)**. **(I,J)** The eight amino acids at the C-terminus of ROXY1 (deleted in CFPN7-ROXY1ΔC129) **(I)** were not necessary to bind with PTL, as joint expression with YFP-PTLmNLS resulted in the latter now occurring preferentially in the nucleus (**J**, compare **E**). Bars represent 50 μm.

First, YFP-ROXY1 fluorescence was shown to be mostly cytoplasmic when expressed alone, although weak nuclear accumulation was sometimes detected (**Figure 5A**). The relative amounts varied somewhat between experiments, perhaps reflecting differing physiological states of the leaves used. The potential partner, PTL, was tagged with CFP that carried an added nuclear localization sequence N7 to ensure nuclear accumulation (Kaplan-Levy et al., 2014) (**Figure 5B**). When YFP-ROXY1 was co-expressed with CFPN7-PTL, YFP fluorescence was now consistently localized to the nuclei (**Figure 5C** compared with **Figure 5A**), as observed in duplicate inoculations over three or more biologically replicated experiments. This was due to the presence of PTL sequences, as controls involving CFPN7 without PTL did not result in nuclear enrichment of YFP-ROXY1 (**Figure 5D**).

To further test this interaction, the converse arrangement was generated. The three NLSs in PTL were mutated resulting in its specific cytoplasmic accumulation (Kaplan-Levy et al., 2014) (**Figure 5E**). On the other hand, ROXY1 was attached to CFPN7, and its fluorescence was now mostly nuclear, although some cytoplasmic fluorescence was still consistently retained (**Figure 5F**). When these two constructs were co-expressed, strong nuclear fluorescence of the previously cytoplasmic YFP-PTLmNLS was seen (**Figure 5G**), presumably through its strong binding with CFPN7-ROXY1. Again, the control CFPN7 alone was ineffective (**Figure 5H**). Thus it seems ROXY1 and PTL sequences can bind each other tightly, and jointly accumulate in nuclei directed by the strong NLS N7.

Finally we tested whether the eight residues at the C-terminal region of ROXY1 are required for interaction with PTL, as they are for binding TGA transcription factors including PERIANTHIA (**Supplementary Figure S4**) (Li et al., 2009). A truncated version lacking these amino acids (CFPN7-ROXY1ΔC129) was generated. Unexpectedly, this consistently resulted in stronger nuclear localization than the full-length version CFPN7-ROXY1 (**Figure 5I** compared with **Figure 5F**), perhaps because a process promoting its cytoplasmic localization was disrupted. Again, co-expression of CFPN7-ROXY1ΔC129 with YFP-PTLmNLS resulted in much higher relative levels of nuclear YFP fluorescence (**Figure 5J**), revealing that the eight C-terminal residues of ROXY1 are not necessary for the interaction with PTL.

### PTL Retains Second-Whorl Function When Three Conserved Cysteines Are Replaced by Serine or Alanine

**FIGURE 6:**
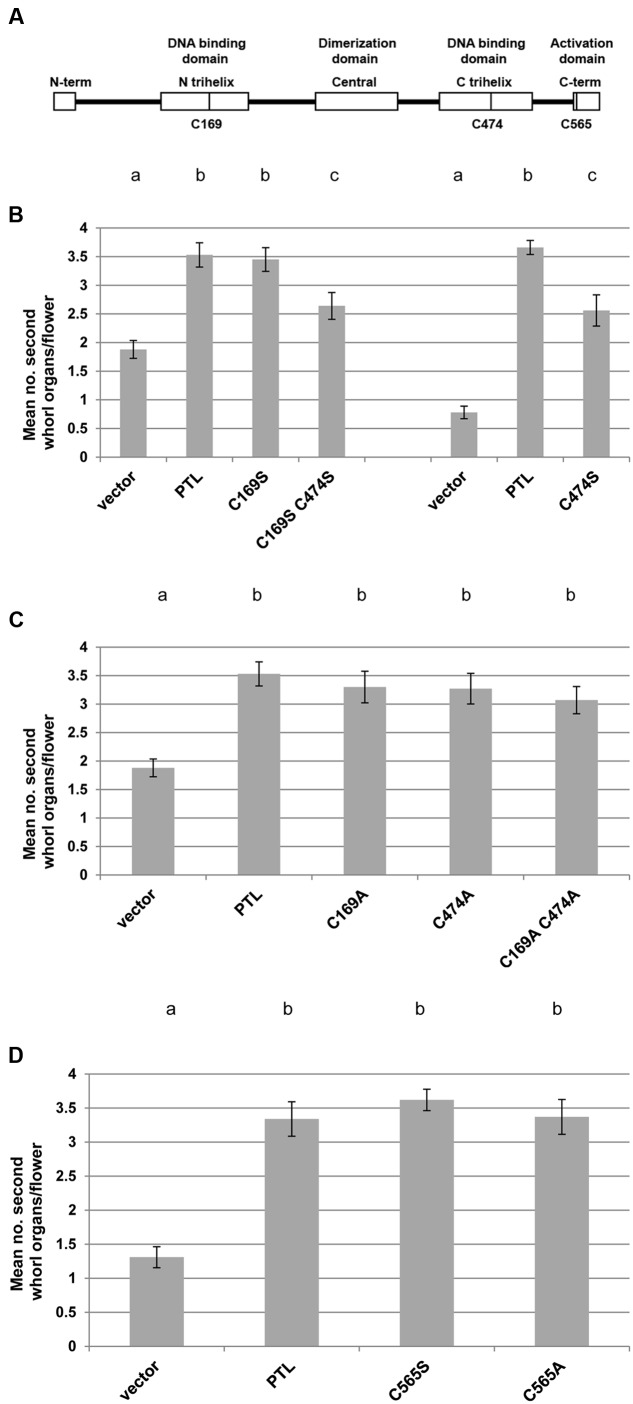
**Complementation tests of second whorl organ numbers in *ptl-1* mutant plants transformed with PTL transgenes carrying cysteine mutations.** **(A)** Sites of three conserved cysteines in the PTL protein that were mutated to either serine or alanine. **(B–D)** Mean number of second whorl organs per flower in *ptl-1* T1 plants transformed with PTL [pPTL(1.3i):2A-GUS-2A-PTL] following site-directed mutagenesis. Negative controls were T1 plants transformed with the empty vector, and positive controls were plants transformed with the wild type PTL. Comparisons were made within individual experiments except for serine mutations of the cysteines in the two DNA binding domains (C169 and C474) which were tested in two separate experiments, with relevant controls in each case **(B)**. Individual data for the 12–15 T1 plants scored for each treatment are provided in **Supplementary Figure S5**. Bars represent SEM. Pairwise comparisons by *t*-tests were carried out. Observations that were not significantly different (*p* > 0.05) are indicated by the same letter at the top of each column within experiments. All other comparisons within experiments were significantly different (*p* < 0.01).

Given that ROXY1 can physically interact with PTL, it may be that it can catalyze reduction of cysteines within the PTL protein to activate it, or otherwise influence its function. A strongly conserved cysteine occurs in each of the duplicated trihelix DNA binding domains (**Figure 6A**) (Kaplan-Levy et al., 2014). Each was mutated individually to either serine or alanine, and double serine mutants and double alanine mutants were also created. Changing cysteine to serine or alanine mimics its reduced form, resulting in a replacement of around the same size but which cannot form disulfide bridges or glutathione adducts. These cysteine mutants were then tested for their ability to complement the loss of petals in *ptl-1* mutant plants.

In most cases cysteine mutations in the DNA binding domains were just as capable of restoring petals as the wild type PTL protein (**Figures 6B,C**; **Supplementary Figures S5A–C**). The only significant reduction occurred when cysteine 474 in the second trihelix DNA binding domain was modified to serine, either alone or in combination with the same change in the first trihelical domain (**Figure 6B**). Even so, complementation very close to a mean of four petals per plant occurred in one or two of the T1 plants, showing that full petal initiation function could still be achieved (**Supplementary Figures S5A,B**). The same losses were not seen when cysteine 474 was changed to alanine (**Figure 6C**; **Supplementary Figure S5C**).

The function of another cysteine conserved in PTL orthologs, C565 in the C terminal activation region (Kaplan-Levy et al., 2014) (**Figure 6A**), was also tested. Again there was no significant reduction in complementation for either serine or alanine mutations (**Figure 6D**; **Supplementary Figure S5D**). Together these results are consistent with all three cysteines occurring in the reduced form in active PTL, although changing the cysteine in the second DNA binding domain to serine reduced PTL function.

## Discussion

In this study, we have shown that PTL and ROXY1 can interact both genetically and at the protein level. Also, PTL can function even if three conserved cysteines are converted to chemically similar amino acids that cannot form disulfide bonds. Thus under conditions of low oxygen it may be that in some circumstances ROXY1 can reduce cysteines in PTL to activate its growth-repressing functions.

### PTL and ROXY1 Interactions Differ in the First and Second Whorls

**FIGURE 7:**
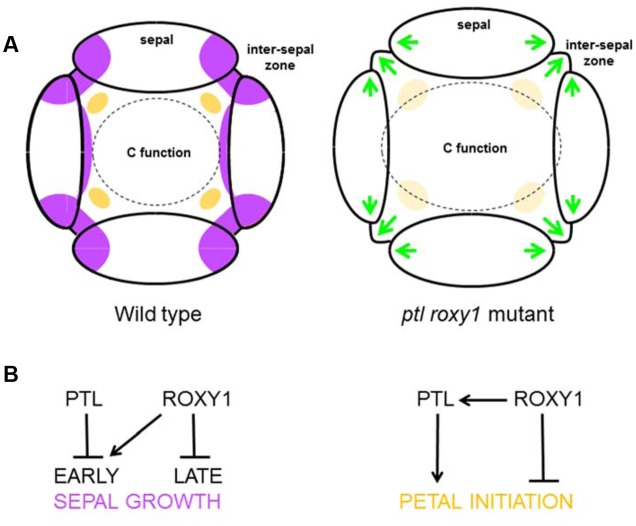
**Proposed functions of PTL and ROXY1 in sepal and petal development.** **(A)** Morphology at stage 4. In the wild type (left), growth in the inter-sepal zones and at the basal margins of sepal primordia is inhibited (purple), and the anlagen of petal primordia are being specified internal to the inter-sepal zone (orange). When both PTL and ROXY1 functions are lost (right), ectopic growth occurs in sepal margins and inter-sepal zones (green arrows). Second whorl organ initiation is not compromised (possibly larger areas compensate for a weaker signal, pale orange), although organ identity is affected by extension of the C-function boundary outward, resulting in significant numbers of stamenoid petals and stamens. **(B)** Proposed gene action. In the first whorl (left), PTL may inhibit early growth of inter-sepal zones and sepal margins where it is normally expressed, whereas ROXY1 may independently promote it. Later ROXY1 may function to inhibit growth. In the second whorl (right), ROXY1 may support petal initiation through activation of PTL, and play an independent inhibitory role (see text).

In the first whorl, loss of PTL results in some overgrowth of inter-sepal zones and the sepals themselves (Griffith et al., 1999; Lampugnani et al., 2013). This was enhanced in *ptl roxy1* double mutants (**Figure 7A**) although there was no effect in *roxy1* mutants alone, suggesting that ROXY1 provides partially redundant function to limit growth. Consistent with this, over-expression of either PTL or ROXY1 results in growth inhibition (Brewer et al., 2004; Wang et al., 2009). ROXY1 may promote early growth but later keep it in check so that there is no net effect in *roxy1* single mutants (**Figure 7B**). PTL may have a strong growth inhibition function, and ROXY1’s role may be revealed only when both ROXY1 and PTL functions are lost.

In the second whorl, however, *roxy1* was epistatic to *ptl* as the number of organs in double mutants was now partially restored from the severe loss seen in *ptl* mutants. Thus the absence of many petals in *ptl* mutants depends on ROXY1 function. This was supported by the further loss of petals seen in *ptl* mutant plants when *ROXY1* was mis-expressed in the *PTL* expression region. In the second whorl, therefore, ROXY1 may directly support PTL’s influence on petal initiation, but also have an independent inhibitory role (**Figure 7B**). In this case, loss of petals in *ptl* mutants would depend on this independent ROXY1 inhibition, and the inhibition would be relieved in *roxy1* mutants whether or not PTL function was present. In *roxy1* single mutants, some reduction in petal number would occur as observed if loss of this independent inhibition was outweighed by the effects of loss of PTL-mediated promotion.

### Loss of PTL and ROXY1 Function May Affect Auxin Dynamics

Even though the developmental processes affected following mutant disruptions in whorls 1 and 2 differ, auxin is involved in both. Disruption of auxin influx into the L1 layer (as in *aux1* mutants) (Reinhardt et al., 2003) had impacts in both whorls. In whorl 1, it was seen only when both PTL and ROXY1 functions were also compromised, in which case further striking ectopic outgrowth of sepals and especially inter-sepal zones occurred. This may be the result of auxin accumulation in the tissues underlying the epidermis, thus promoting additional organ growth already sensitized by the loss of PTL and ROXY1 functions.

In whorl 2, *roxy1 aux1* double mutants lost additional second whorl primordia, suggesting that auxin signaling is also affected in *roxy1* single mutants as we have previously shown for *ptl* (Lampugnani et al., 2013). Nevertheless, in *ptl roxy1 aux1* triple mutants the further loss of AUX1 function did not have a major second whorl effect as organ numbers were maintained at the *ptl roxy1* level. It seems that when both PTL and ROXY1 functions are compromised there is sufficient auxin signal in whorl 2 to support most organ initiation, whether or not there is movement of auxin into the epidermis by AUX1. The area occupied by whorl 2 in *ptl roxy1* double mutants is increased (at least in mature flowers), so perhaps there is a larger region capable of petal initiation in stage 4 buds.

Thus in the wild type the three proteins PTL, ROXY1, and AUX1 may limit auxin function in the first whorl between and within sepal primordia, keeping growth in check. Conversely, they may promote auxin function in the second whorl supporting the initiation of petal primordia. These roles may be associated with the proposed temporal movement of auxin from the first to the second whorl during early flower development (Lampugnani et al., 2013). AUX1 is not involved in such lateral auxin transport, but the polar auxin transport proteins PIN1 and PID are, and each promotes petal initiation (presumably through auxin accumulation) in the second whorl (Lampugnani et al., 2013).

It should be emphasized that the involvement of auxin revealed here could be the secondary consequence of growth disruptions that result from the loss of PTL or ROXY1 functions. At this stage there is no evidence that genes controlling auxin biosynthesis, transport or signaling are the direct targets of PTL regulation, or that such proteins are subject to disulphide reduction or deglutathionylation by ROXY1, and future tests of such events are required.

### C Function Is Jointly Repressed by PTL and ROXY1 in the Outer Whorls

The identity of second whorl organs was often modified in *ptl-1 roxy1-3* double mutant flowers. More than one third were now stamens or stamenoid petals, implying extension of AGAMOUS (AG)-directed C function outward into the second whorl (**Figure 7A**). Ectopic carpelloid outgrowths also occurred on sepal margins in ∼5% of flowers indicating C function activity in the first whorl as well. In both whorls the effects were magnified upon loss of AUX1 function.

Similar ectopic extension of C function was also seen when *roxy1* mutants were combined with other mutations (Xing et al., 2005), including mutations of the zinc finger repressor gene *RABBIT EARS* (*RBE*). Loss of RBE function alone resulted in loss of some petals (Takeda et al., 2004), and also some AG mis-expression and organ identity defects (Krizek et al., 2006), and these disruptions were enhanced when RBE and ROXY1 function were conjointly compromised (Xing et al., 2005). PTL may act in a common developmental pathway with RBE as their loss of function phenotypes overlap, and there is no further loss of second whorl organs in *ptl-1 rbe-3* double mutant flowers (Lampugnani et al., 2013). Their responses to disruptions in auxin dynamics are also similar.

The mechanistic basis of this ectopic C function is not clear, although disruptions to boundaries in the floral meristem may be involved. RBE is involved in organ boundary specification as it directly represses expression of *EXTRA EARLY PETALS1* (*EEP1*), a microRNA that negatively regulates two *CUC* boundary genes *CUC1* and *CUC2* (Huang et al., 2012). Mutants of *ptl* also interact genetically with *eep1* and *cuc1* mutants in the outer whorls (Lampugnani et al., 2012), although PTL is a transcriptional activator not a repressor (Kaplan-Levy et al., 2014), and its direct targets have not been established.

Interestingly, PAN, a partner of ROXY1, binds to a regulatory element in the intron of *AG* and promotes its expression (Das et al., 2009; Maier et al., 2009). Further, such binding occurs *in vitro* only under reducing conditions (Gutsche and Zachgo, 2016). However, direct regulation of *AG* expression by PAN is likely to be limited to the fourth whorl (Das et al., 2009), imposing determinacy at the center of the floral meristem (Maier et al., 2009), and thus independent of repression of *AG* in the second whorl.

### Inter-sepal Boundary Zones Retain Meristematic Potential

One striking finding was that ectopic outward growth from inter-sepal zones occurred when PTL, ROXY1, and AUX1 function were simultaneously lost. Presumably such growth is normally suppressed to maintain inter-organ space and organ separation. Here, we have shown that these boundary regions retain their ability to grow outward, and even to generate stalked structures and aggregates of sepal-like organs. It may be that such organ clusters represent a partial triggering of the flower development program, and it will be informative to test if expression of flower identity genes *LEAFY* and *APETALA1* (Weigel and Meyerowitz, 1993) is ectopically induced in this region. Loss of AUX1 function might promote such outgrowths by allowing auxin to accumulate sub-epidermally, where it might trigger or promote meristematic growth. This could also be tested, in this case using reporters of auxin function. The inter-sepal zone may be a region of low oxygen concentration that ROXY1 somehow reacts to and supports the growth-repressing function of PTL. PTL also binds the low energy-sensing kinase AKIN10 component of SnRK1 (O’Brien et al., 2015), and the combined action of ROXY1 and AKIN10 might reinforce growth suppression through their independent activation of PTL in hypoxic regions with low energy levels.

### Physical Interaction of PTL and ROXY1 Proteins

As well as interacting genetically, PTL and ROXY1 proteins can bind when transiently co-expressed in leaves. This needs to be examined by additional methods such as co-immunoprecipitation tests *in vitro* and *in vivo*, and in yeast expression systems, but if confirmed, how might a direct interaction occur in developing buds? Clearly the two proteins need to be present in the same cell. However, their gene expression patterns at stages 3 and 4 do not overlap directly. *PTL* is expressed in inter-sepal zones from stage 3 and in sepal margins from stages 4–5, whereas *ROXY1* is expressed in organ anlagen and in the tips of newly arising organs (**Supplementary Figure S1**). There is no evidence that PTL mRNA or protein moves between cells, and accumulation of pROXY1:ROXY1-GFP fusion protein matches the sites of accumulation of ROXY1 mRNA (Xing et al., 2005), indicating that it does not move. Even so, ROXY1 is a relatively small 14.2 kDa protein that may be able to move between cells without the GFP tag. Also PTL may be a long-lived protein that continues to occupy the petal initiation region derived from the earlier strong PTL expression domain at stage 3. This could be tested using PTL tagged with long-lived fluorescent timers for example (Subach et al., 2009).

PETAL LOSS is nuclear localized (Kaplan-Levy et al., 2014), but ROXY1 occurs variably in both nucleus and cytoplasm, although its petal functions depend on its presence in the nucleus (Li et al., 2009). Here, we showed that ROXY1 occurred preferentially in the nucleus following its association with PTL in transient expression assays. This is consistent with their physical association having functional significance, although this will require further experimental testing.

ROXY1 interacts with TGA transcription factors at its C-terminal region. Deletion of the eight C-terminal amino acids of ROXY1 disrupts binding to PAN and other TGA transcription factors (Li et al., 2009). The deletion apparently disrupts an α-helical region that extends into the adjacent conserved motif LXXLL which provides the specificity needed for the ROXY1–PAN interaction and is essential for ROXY function (Li et al., 2011). The 8-residue deletion includes the ALWL motif at the very C-terminus of ROXY1 also required for its petal development function but not TGA interaction (Li et al., 2009). On the other hand, we found that deletion of these eight amino acids of ROXY1 did not affect PTL binding (although it did boost the amount of ROXY1 occurring in the nucleus). The active site of ROXY1 lies internal to this region, so conceivably it could modify the thiol groups of both PAN and PTL, although the ROXY1 domains that bind them may differ. Alternatively, different accessory proteins may be involved.

### PTL As a Potential Target for ROXY1 Reduction

If PTL is a target of ROXY1, it may be active only if specific cysteine residues are in the reduced state. Each of the duplicated DNA binding domains of PTL has a cysteine at a position conserved throughout trihelix proteins (Nagano, 2000; Kaplan-Levy et al., 2014). We changed these to alanine or serine to chemically mimic the reduced state of cysteine, and found that the PTL protein was still functional. There was no detectable effect when either or both cysteines were replaced by alanine, a slightly smaller but still hydrophobic amino acid. When changed to serine, similar in size to cysteine but more hydrophilic, a change in the N-terminal trihelix also had no major effect but a C-terminal change resulted in a significant reduction, although not full loss, of function.

The trihelix DNA binding domain is distantly related to that of MYBs, and conserved cysteines occur at equivalent positions (Nagano, 2000). Our findings match those in c-Myb of humans where mutation of the conserved cysteine to alanine in the R2 repeat did not affect DNA binding *in vitro*, or its function *in vivo*, but a change to serine decreased both (Myrset et al., 1993). In c-Myb this region undergoes a conformational change when it binds DNA, with the cysteine now present internally, and folding is apparently blocked if the cysteine is changed to serine but not alanine. Similar differential disruptions to DNA binding have been reported for cysteine substitutions in the P1 Myb of maize (Heine et al., 2004). The same might be the case in PTL, as the solution structure of a trihelix relative GT-2 indicates that the conserved cysteine occupies a similar internal position (Nagata et al., 2010). Why substitution in the N-terminal trihelix of PTL does not significantly reduce function is not known, although the two trihelix domains are relatively divergent (Kaplan-Levy et al., 2014). Conserved cysteine 565 in the PTL activation domain was also tested and could be changed to either alanine or serine without impacting on its petal initiation role. Overall, our results are consistent with PTL functioning when the three conserved cysteines are in the reduced state, although biochemical studies are needed to further test this, and to determine if their oxidation blocks PTL function.

### Wider Developmental Roles of ROXY Glutaredoxins and Trihelix Transcription Factors

In addition to petal initiation and growth, developmental functions of ROXY GRXs include control of sporogenous cell identity in anthers of Arabidopsis (Xing and Zachgo, 2008), rice (Murmu et al., 2010; Hong et al., 2012), and maize (Kelliher and Walbot, 2012). In Arabidopsis, *ROXY1* functions redundantly in this with its close paralog *ROXY2* (Xing and Zachgo, 2008) and it will be interesting to see if the two genes also act redundantly in perianth development.

Recently, it has been revealed that the maize *Male sterile converted anther* (*Msca1*) GRX gene is also involved in supporting the size of the shoot apical meristem (Yang et al., 2015). It does this by negatively regulating the size-restraining influence of Fasciated ear4 (Fea4) (Paulter et al., 2015). Fea4 is the maize ortholog of PAN of Arabidopsis, so a functional relationship between specific ROXY and TGA proteins extends to the shoot as well as the flower meristem. This is consistent with evidence that PAN can influence organ numbers in flowers by restricting the size of the flower meristem (Maier et al., 2009).

There are 30 trihelix genes in Arabidopsis, and their functions have diversified to include regulation of developmental processes, and responses to biotic and abiotic stresses (Kaplan-Levy et al., 2012). In this regard, they parallel the diversity of developmental and stress-related functions seen among members of the ROXY clade (Gutsche et al., 2015). ROXY functional variation is also matched by division of labor among those TGA transcription factors that interact specifically with each functional group. ROXY1 and PTL are both involved in perianth development, and it will be of interest to test if other trihelix proteins interact with different members of the ROXY family in a parallel, functionally related pattern.

## Author Contributions

TQ conceived the study, performed all experiments except SEM, interpreted the results, and prepared drafts of the manuscript. EL provided cloning vectors and performed the SEM experiments, discussed results, and helped prepare the manuscript. DS provided overall supervision and funding, helped interpret results, and prepared the manuscript with the assistance and approval of the other authors.

## Conflict of Interest Statement

The authors declare that the research was conducted in the absence of any commercial or financial relationships that could be construed as a potential conflict of interest.
