# Supplementary material for: PETAL LOSS and ROXY1 Interact to Limit Growth Within and between Sepals But to Promote Petal Initiation in Arabidopsis thaliana
Source: Front Plant Sci. 2017 Feb 8;8:152. doi: 10.3389/fpls.2017.00152 (PMC5296375; doi:10.3389/fpls.2017.00152)
Supplement: Supplementary file 1 [file Data_Sheet_1.pdf]

Supplemental Material

PETAL LOSS and ROXY1 interact to limit growth within and between sepals but to promote petal initiation in *Arabidopsis thaliana*

Tezz Quon, Edwin R Lampugnani and David R Smyth

Table S1. Primers used in the study.

Table S2. Mean number of floral organs per flower in wild type and *ptl*, *roxy1* and *aux1* single and multiple mutants

Table S3. Mean number and form of second whorl organs per flower in wild type and *ptl*, *roxy1* and *aux1* mutants

Table S4. Mean number of third whorl stamens per flower in wild type and *ptl*, *roxy1* and *aux1* mutants

Table S5. Mean number of outgrowths of various types per flower on the edge of first whorl sepals in wild type and *ptl*, *roxy1* and *aux1* mutants

Table S6. Mean number of clusters of ectopic sepals per flower arising from the inter-sepal zone in wild type and *ptl*, *roxy1* and *aux1* mutants

Figure S1. Expression patterns of *PTL* and *ROXY1* in buds of Arabidopsis

Figure S2. Second whorl organ numbers in individual T1 transgenic plants transformed with pPTL:ROXY1.

Figure S3. Outgrowth of inter-sepal zones in mature siliques of wild type and *ptl-3* mutant plants.

Figure S4. C-terminal amino acid sequences of ROXY1 and ROXY1ΔC129

Figure S5. Second whorl organ numbers in individual *ptl-1* T1 transgenic plants transformed with cysteine mutants of PTL.

Table S1. Primers used in the study.

Key: S - sense strand; A - antisense strand; f- forward; r- reverse; bold - restriction site; underlined - start or stop; double underlined – site-directed mutations.

Name	Sequence
ptl-1 genotyping (*Hae*III digest of *PTL* 138bp + 390bp; *ptl-1* 528bp)	
S-ptl-30F P1 f	5'-TCTCTTTCGGCTGAATATAAATCTG-3'
A-ptl+498R P1 r	5'-ATTAGGGTAAAATCGAGAGAGGAGA-3'
roxy1-3 genotyping (T-DNA insertion mutant)	
roxy1-3 o8474 LB	5'-ATAATAACGCTGCGGACATCTACATTTT-3'
A-ROXY1 +143 r	5'-GTGCTCACGCTGAAGATCAC-3'
aux1-21 genotyping (*Apa*LI digest of *AUX1* 443bp; *aux1-21* 220bp + 223bp)	
S-AUX1 +600 f	5'-CATTTAATTGCACTTTCCTCTTGTT-3'
A-AUX1 +1043 r	5'-CATATACTGTCACCTCAATGCAAAG-3'
CFPN7	
A-N7 No Stop EcoRI	5'-CTCT**GAATTC**CTCTTCTTCTTGATCAGCTTC-3'
S-XhoI ATG N7 f	5'-CGAG**CTCGAG**ATGTTCAAGCGTGAAGAGCAAGC-3'
A-SalI No Stop CFP r	5'-ATAT**GTCGAC**CTTGTACAGCTCGTCCATG-3'
S-XhoI ATG CFP f	5'-ATAT**CTCGAG**ATGGTGAGCAAGGGCG-3'
ROXY1 coding sequence	
S-*Eco*RI ATG ROXY1 f	5'-ATAT**GAATTC**ATGCAATACCAGACAGAATCG-3'
S-*Kpn*I ATG ROXY1 f	5'-ATAT**GGTACC**ATGCAATACCAGACAGAATCG-3'
A-ROXY1 Stop *Bam*HI r	5'-ATAT**GGATCC**TCAGAGCCAGAGAGCG-3'
A-ROXY1ΔC129 Stop *Bam*HI r	5'-ATAT**GGATCC**TCAGAGAAGAGGAACGAGAGA-3'
PTL coding sequence	
S-*Eco*RI-PTL f	5'-C**GAATTC**ATGGATCAAGATCAGCAT-3'
A-PTL-Stop-*Bam*HI r	5'-CG**GGATCC**TTACTGATTCTCTTCTTT-3'
pPTL promoter GUS (pPTL(1.3i):2A-GUS-2A)	
S-*Nsi*I pPTL(1.3i) 5' f	5'-ATTA**ATGCAT**CAATGAATGTCCGAACGGTA-3'
A-pPTL(1.3i) *Asc*I 3' r	5'-ATAT**GGCGCGCC**CATAATCCTATTTAAGAATATAATAATAAAAG-3'
A-*Xho*I GUS ATG r	5'-GTG**CTCGAG**ATGGTCCGTCCTGTAGAAAC-3'
S-GUS no stop *Asc*I f	5'-GG**GGCGCGCC**TTGTTTGCCTCCCTGCTGCG-3'
S-GUS +1550 f screening	5'-ACACCGACATGTGGAGTGAAG-3'

A-3' OCS r screening	5'-CATGCGATCATAGGCGTCTC-3'
S-*Asc*I 2A f frame	5'-CG**GGCGCGCC**CGGCAGTGGAGAGGGCAGAGGA-3'
A-2A 3' *Sal*I r	5'-GG**GTCGAC**TGGGCCAGGATTCTCCTC-3'
S-*Asc*I 2A *Sal*I f template	5'CG**GGCGCGCC**CGGCAGTGGAGAGGGCAGAGGAAGTCTGCTAAC ATGCGGTGACGTCGAGGAGAATCCTGGCCCA**GTCGAC**CC-3'
A-2A 3' *Kpn*I r	5'-GG**GGTACC**TGGGCCAGGATTCTCCTC-3'
Site-directed mutagenesis of cysteines in PTL	
S-ptl C169S f	5'-GGAAGAAATCTAGAGAGAAGTTTG-3'
S-ptl C169A f	5'-GGAAGAAAGCTAGAGAGAAGTTTG-3'
A-ptl C169S r	5'-TTCTCTCTAGATTCTTCCCACTC-3'
A-ptl C169A r	5'-TTCTCTCTAGCTTCTTCCCACTC-3'
S-ptl C474S f	5'-CCTTATTATCTAAGGAAAAGTGGG-3'
S-ptl C474A f	5'-CCTTATTAGCTAAGGAAAAGTGGG-3'
A-ptl C474S r	5'-TTTTCCTTAGATAATAAGGCACTT-3'
A-ptl C474A r	5'-TTTTCCTTAGCTAATAAGGCACTT-3'
S-ptl C565S f	5'-GTACAAACTCTTTCCCGTTCTTCAGT-3'
S-ptl C565A f	5'-GTACAAACGCTTTCCCGTTCTTCAGT-3'
A-ptl C565S r	5'-AACGGGAAAGAGTTTGTACTAGCAG-3'
A-ptl C565A r	5'-AACGGGAAAGCGTTTGTACTAGCAG-3'

Table S2. Mean number of floral organs per flower ± standard error in wild type and *ptl*, *roxy1* and *aux1* single and multiple mutants.

	Columbia	*ptl-1*	*roxy1-3*	*ptl-1 roxy1-3*	*aux1-21*	*ptl-1 aux1-7*	*roxy1-3 aux1-21*	*ptl-1 roxy1-3 aux1-21*
First whorl*	4.00 ± 0.000	4.00 ± 0.000	4.00 ± 0.018	4.00 ± 0.000	4.00 ± 0.000	4.03 ± 0.021	4.00 ± 0.000	3.99 ± 0.013
Second whorl	4.00 ± 0.000	2.08 ± 0.195	3.64 ± 0.077	3.45 ± 0.178	3.67 ± 0.088	0.00 ± 0.000	1.66 ± 0.133	3.87 ± 0.170
Third whorl	5.89 ± 0.048	6.12 ± 0.041	5.23 ± 0.091	5.15 ± 0.095	5.65 ± 0.078	5.66 ± 0.080	4.49 ± 0.125	4.22 ± 0.178
Fourth whorl	2.00 ± 0.000	2.00 ± 0.000	2.00 ± 0.000	2.00 ± 0.000	2.00 ± 0.000	2.00 ± 0.000	2.00 ± 0.000	2.00 ± 0.000
Total no. of flowers	79	78	88	78	87	78	76	78

* excluding clusters of ectopic sepals generated from the extended inter-sepal zone

Table S3. Mean number and form of second whorl organs per flower ± standard error in wild type and *ptl*, *roxy1* and *aux1* mutants.

	Columbia	*ptl-1*	*roxy1-3*	*ptl-1 roxy1-3*	*aux1-21*	*ptl-1 aux1-7*	*roxy1-3 aux1-21*	*ptl-1 roxy1-3 aux1-21*
Normal petals	3.99 ± 0.013	1.00 ± 0.121	2.69 ± 0.121	0.58 ± 0.096	3.60 ± 0.096	0.00 ± 0.000	0.70 ± 0.104	0.40 ± 0.074
Small/folded petals	0.01 ± 0.013	0.36 ± 0.073	0.90 ± 0.090	1.15 ± 0.114	0.07 ± 0.047	0.00 ± 0.000	0.92 ± 0.099	0.87 ± 0.095
Tubular petals	0.00 ± 0.000	0.22 ± 0.054	0.01 ± 0.013	0.05 ± 0.025	0.00 ± 0.000	0.00 ± 0.000	0.00 ± 0.000	0.00 ± 0.000
Filamentous organs	0.00 ± 0.000	0.40 ± 0.076	0.03 ± 0.018	0.29 ± 0.073	0.00 ± 0.000	0.00 ± 0.000	0.04 ± 0.029	0.13 ± 0.042
Sepaloid petals	0.00 ± 0.000	0.00 ± 0.000	0.01 ± 0.013	0.12 ± 0.045	0.00 ± 0.000	0.00 ± 0.000	0.00 ± 0.000	0.18 ± 0.047
Stamenoid petals	0.00 ± 0.000	0.05 ± 0.025	0.00 ± 0.000	0.78 ± 0.101	0.00 ± 0.000	0.00 ± 0.000	0.00 ± 0.000	1.32 ± 0.120
Stamens	0.00 ± 0.000	0.05 ± 0.025	0.00 ± 0.000	0.45 ± 0.089	0.00 ± 0.000	0.00 ± 0.000	0.00 ± 0.000	0.97 ± 0.095
Total	4.00 ± 0.000	2.08 ± 0.195	3.64 ± 0.077	3.45 ± 0.178	3.67 ± 0.088	0.00 ± 0.000	1.66 ± 0.133	3.87 ± 0.170
Total no. of flowers	79	78	88	78	87	78	76	78

Table S4. Mean number of third whorl stamens per flower ± standard error in wild type and *ptl*, *roxy1* and *aux1* single and multiple mutants.

Number of third whorl stamens per flower	Columbia	*ptl-1*	*roxy1-3*	*ptl-1 roxy1-3*	*aux1-21*	*ptl-1 aux1-7*	*roxy1-3 aux1-21*	*ptl-1 roxy1-3 aux1-21*
0							1	3
1								2
2							3	5
3			1	1			2	11
4	2		15	17	4	4	34	22
5	6		25	31	13	13	23	14
6	70	70	47	27	70	61	13	21
7	1	7		2				
8		1						
Total no. of flowers	79	78	88	78	87	78	76	78
Mean no. per flower ± SE	5.89 ± 0.048	6.12 ± 0.041	5.23 ± 0.091	5.15 ± 0.095	5.65 ± 0.078	5.66 ± 0.080	4.49 ± 0.124	4.22 ± 0.178
Mean no. medial stamens	4.01 ± 0.001	4.09 ± 0.004	3.99 ± 0.001	3.86 ± 0.044	4.00 ± 0.000	3.82 ± 0.007	3.81 ± 0.008	3.01 ± 0.137
Mean no. lateral stamens	1.87 ± 0.005	2.03 ± 0.002	1.28 ± 0.009	1.29 ± 0.090	1.63 ± 0.007	1.88 ± 0.004	0.72 ± 0.011	1.21 ± 0.092

Table S5. Mean number of outgrowths of various types per flower ± standard error on the edge of first whorl sepals in wild type and *ptl*, *roxy1* and *aux1* single and multiple mutants.

Type of sepal outgrowth	Columbia	*ptl-1*	*roxy1-3*	*ptl-1 roxy1-3*	*aux1-21*	*ptl-1 aux1-7*	*roxy1-3 aux1-21*	*ptl-1 roxy1-3 aux1-21*
Sepal tissue	0	0	0	0.244 ± 0.069	0	0.051 ± 0.025	0	0.269 ± 0.081
Petal tissue	0	0	0	0	0	0	0	0.051 ± 0.031
Stamen tissue	0	0.026 ± 0.018	0	0	0	0.026 ± 0.026	0	0.013 ± 0.013
Carpel tissue (stigmata, ovules)	0	0.038 ± 0.022	0	0.054 ± 0.111	0	0.038 ± 0.022	0	0.615 ± 0.145
Total no. of flowers	79	78	88	78	87	78	76	78

Table S6. Mean number of clusters of ectopic sepals per flower ± standard error arising from the inter-sepal zone in wild type and *ptl*, *roxy1* and *aux1* mutants.

No. of sepal clusters per flower	Columbia	*ptl-1*	*roxy1-3*	*ptl-1 roxy1-3*	*aux1-21*	*ptl-1 aux1-7*	*roxy1-3 aux1-21*	*ptl-1 roxy1-3 aux1-21*
0	0	0	0	64	0	0	0	40
1	0	0	0	11	0	0	0	21
2	0	0	0	2	0	0	0	11
3	0	0	0	1	0	0	0	5
4	0	0	0	0	0	0	0	1
Total no. of flowers	79	78	88	78*	87	78	76	78**
Mean no. per flower ± SE	0	0	0	0.231 ± 0.063	0	0	0	0.795 ± 0.113

* 18 sepal clusters from 316 inter-sepal zones (5.77%); low variation, ranging from 1 to 6 in the 10 flowers scored for each plant

** 62 sepal clusters from 315 inter-sepal zones (19.94%); high variation, ranging from 2 to 22 in the 10 flowers scored for each plant

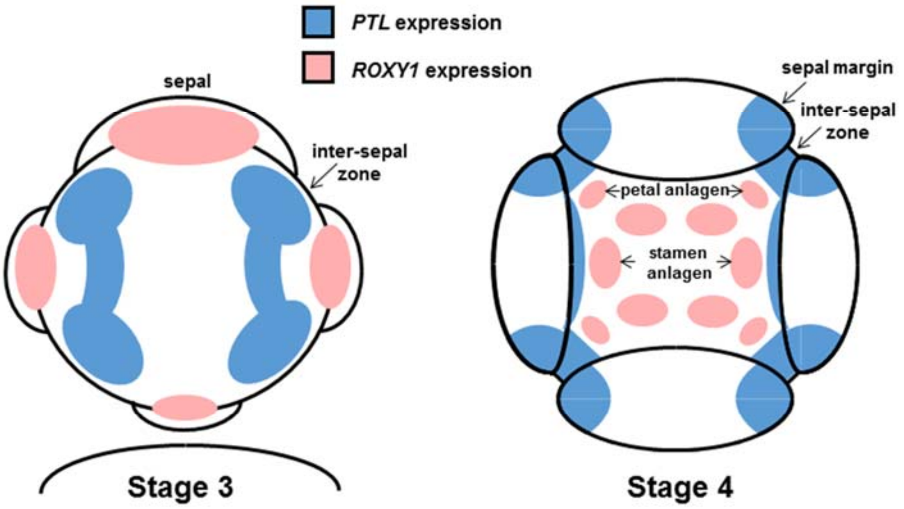

Figure S1. Expression patterns of *PTL* and *ROXY1* in buds of *Arabidopsis*.

Expression of *PTL* (blue) and *ROXY1* (pink) at stages 3 and 4 of flower development (Smyth et al., 1990). *PTL* results are adapted from Brewer et al., (2004) and Lampugnani et al., (2012), and *ROXY1* results are based on Xing et al., (2005), Xing and Zachgo (2008), and Li et al., (2009). *AUX1* is expressed throughout the epidermis (L1) of developing flowers (Reinhardt et al., 2003; Lampugnani et al., 2013).

A.

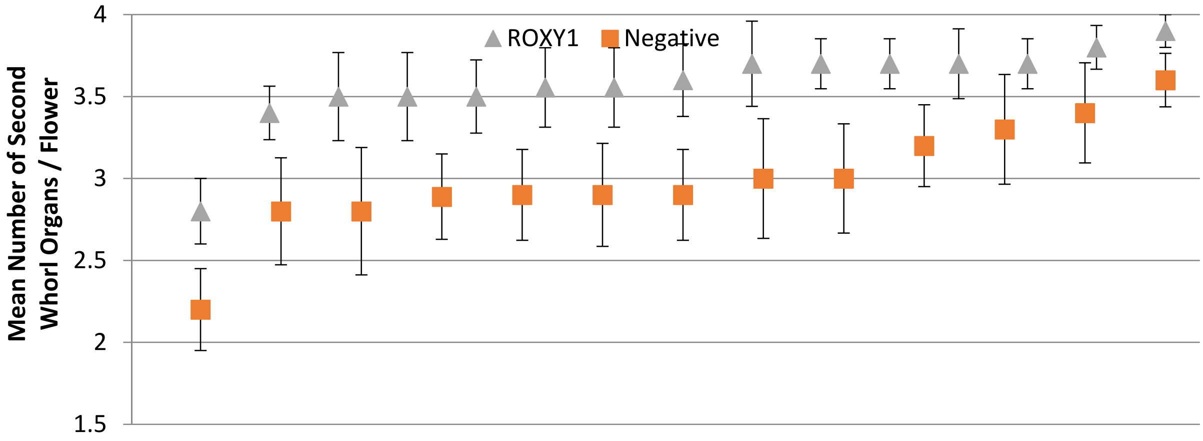

B.

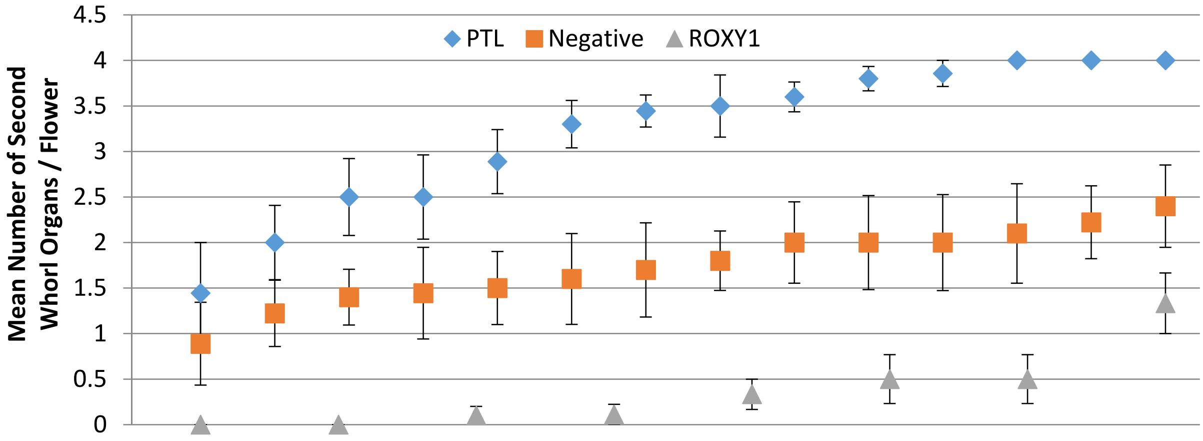

Figure S2. Second whorl organ numbers in individual T1 transgenic plants transformed with pPTL:ROXY1.

The first 10 flowers in T1 plants were scored, and the mean and standard errors per flower
for each plant are shown, ordered from lowest to highest along the Y axis.
Expression of the insert in all constructs was controlled by pPTL(1.3i):2A-GUS-2A.

(A) *roxy1-3* mutants transformed with ROXY1 (positive control) or the empty vector (negative control).
(B) *ptl-1* mutants transformed with ROXY1, the empty vector (negative control) or PTL (positive control).

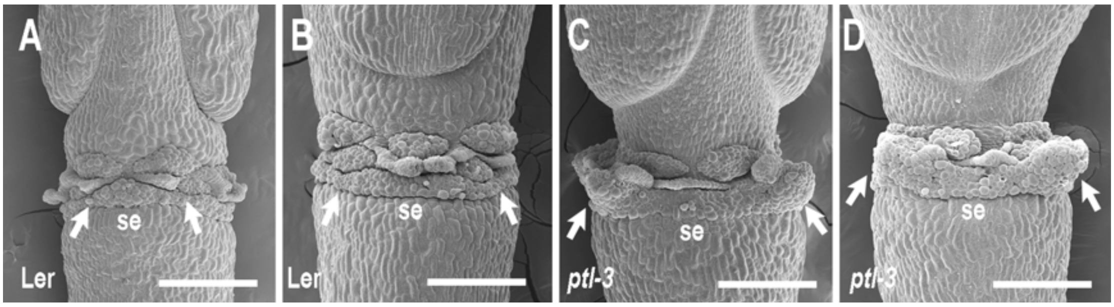

Figure S3. Outgrowth of inter-sepal zones in mature siliques of wild type and *ptl-3* mutant plants.

SEMs of the base of mature stage 17 siliques in lateral (A and C) or medial (B and D) view, showing significant outgrowth of the inter-sepal zones in *ptl-3* mutant flowers (arrows in C and D) compared with wild type Landsberg *erecta* controls (arrows in A and B). Sites of sepal abscission are labelled below (se). Scars where other floral organs have abscised, and swollen nectary tissue, are also present. Bars represent 250 μm.

```
                  120         130    136
                   |     αC    |      |
ROXY1           …INGSLVPLLKDAGALWL*
ROXY1ΔC129      …INGSLVPLL*
```

Figure S4. C-terminal amino acid sequences of ROXY1 and ROXY1ΔC129.

ROXY1ΔC129 lacks the 8 C-terminal amino acids and results in much-reduced binding with TGA transcription factors (Li et al., 2009; 2011). This is likely because it disrupts a C-terminal α–helical region, although site-directed mutagenesis reveals that the leucines in the conserved LXXLL motif (double underline) that remains are specifically required for binding of ROXY1 with PAN and other TGA transcription factors (Li et al., 2011). The conserved C-terminal ALWL motif (dotted underline) is required for ROXY1 function in petal initiation but not for TGA binding (Li et al., 2011).

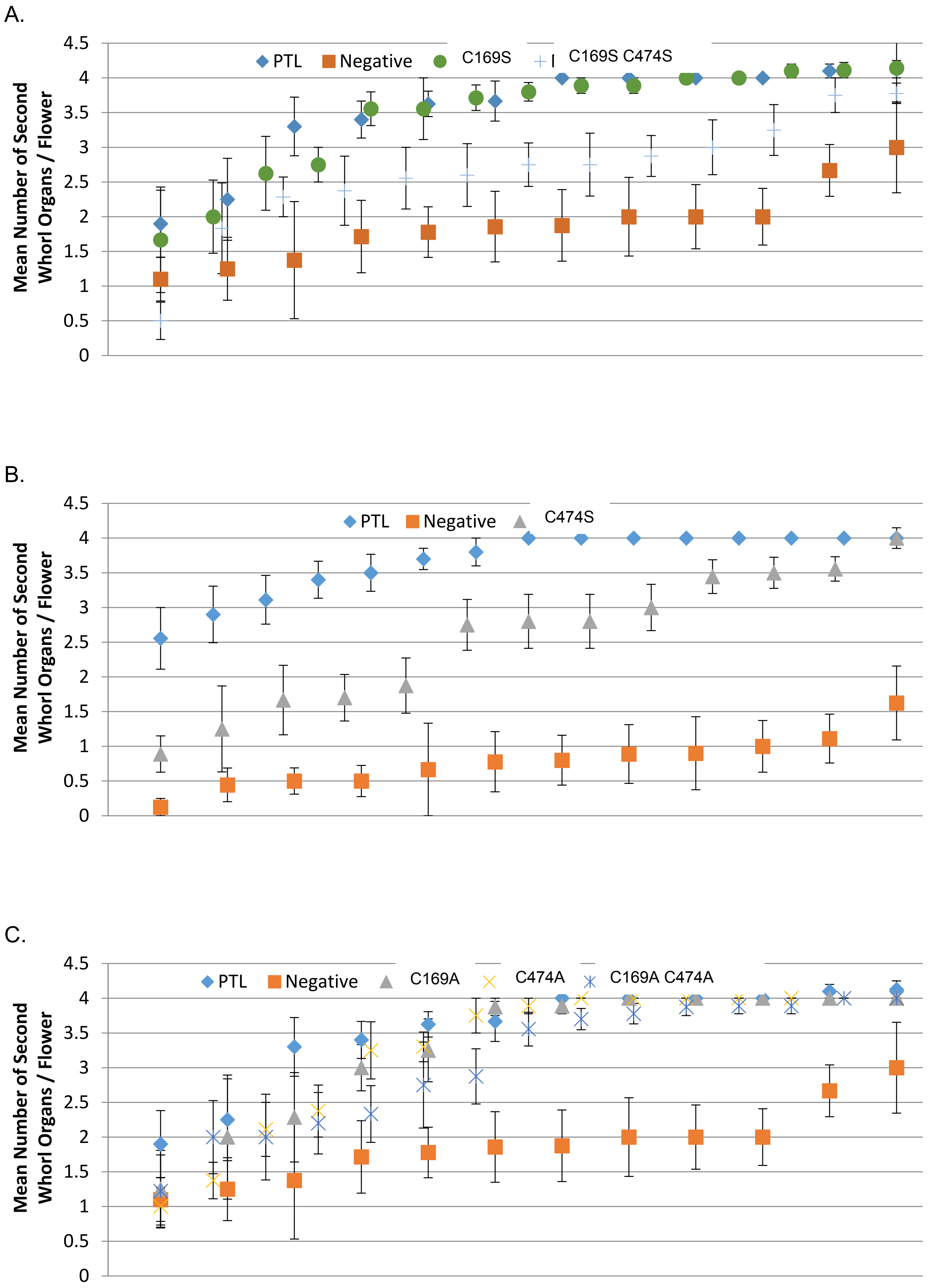
A.
Mean Number of Second Whorl Organs / Flower
4.5
4
3.5
3
2.5
2
1.5
1
0.5
0
PTL
Negative
C169S
C169S C474S
B.
Mean Number of Second Whorl Organs / Flower
4.5
4
3.5
3
2.5
2
1.5
1
0.5
0
PTL
Negative
C474S
C.
Mean Number of Second Whorl Organs / Flower
4.5
4
3.5
3
2.5
2
1.5
1
0.5
0
PTL
Negative
C169A
C474A
C169A C474A

D.

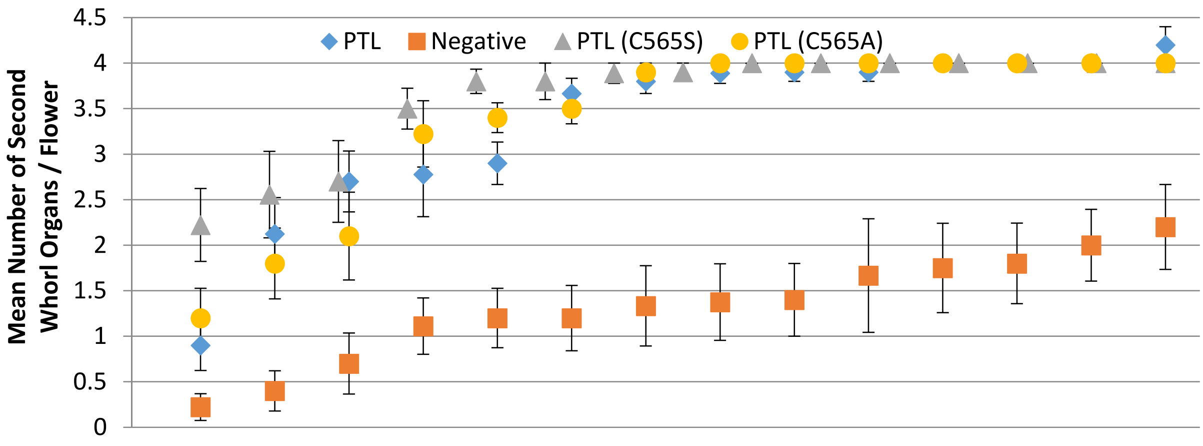

Figure S5. Second whorl organ numbers in individual *ptl-1* T1 transgenic plants transformed with cysteine mutants of PTL.

The first 10 flowers in T1 plants were scored, and the mean and standard errors per flower for each plant are shown, ordered from lowest to highest along the Y axis. Positive controls with wild type PTL, and negative controls with empty vector (pPTL(1.3i):2A-GUS-2A), are included.

(A) Serine mutations of the conserved cysteine in the N-terminal trihelix DNA binding domain, singly (C169S) or in combination with an equivalent mutant in the C-terminal domain (C169S C474S).
(B) Serine mutation of the conserved cysteine in the C-terminal trihelix DNA binding domain (C474S). This was scored with controls in a separate experiment from the other serine substitutions in (A).
(C) Alanine mutations of the conserved cysteines in the N-terminal and C-terminal trihelix DNA binding domains, singly (C169A and C474A) or in combination (C169A C474A)).
(D) Mutations of the conserved cysteine in the C-terminal activation region to serine (C565S) or alanine (C565A).
